# Erratum: Functional environmental genomics of a municipal landfill soil

**DOI:** 10.3389/fgene.2013.00069

**Published:** 2013-04-25

**Authors:** Dick Roelofs

**Affiliations:** Department of Ecological Science, VU UniversityAmsterdam, Netherlands

In Figure 2

An error has been identified in the legend of Figure 2 after our article was published in *Frontiers in Genetics*. The current description in the legend of Figure 2 wrongly assigns red dots to soil samples and blue dots to extract samples.

The correct description in the legend of Figure 2 is as follows:

**Figure 2:**
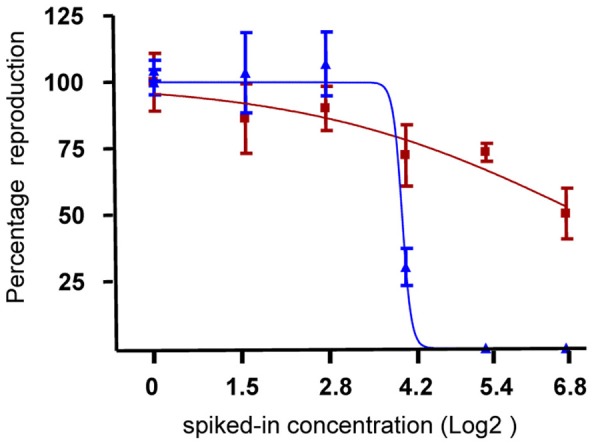
**Effect of soil and extract on reproduction of *F. candida*. Blue** dots indicate the number of *F. candida* juveniles in the jars after 28 days exposure to 6 dilutions of soil samples. **Red** dots indicate the number of juveniles retrieved after 28 days exposure to dilutions of extract samples. The lines indicate the dose-response curves derived from a logistic model. X-axis, Log2 transformed spiked-in concentrations; y-axis, percentage reproduction scaled to the control samples (set at 100%).

